# Machine learning in patient flow: a review

**DOI:** 10.1088/2516-1091/abddc5

**Published:** 2021-02-22

**Authors:** Rasheed El-Bouri, Thomas Taylor, Alexey Youssef, Tingting Zhu, David A Clifton

**Affiliations:** 1 Institute of Biomedical Engineering, University of Oxford, Oxford, United Kingdom

**Keywords:** patient flow, deep learning, machine learning, hospital resource

## Abstract

This work is a review of the ways in which machine learning has been used in order to plan, improve or aid the problem of moving patients through healthcare services. We decompose the patient flow problem into four subcategories: prediction of demand on a healthcare institution, prediction of the demand and resource required to transfer patients from the emergency department to the hospital, prediction of potential resource required for the treatment and movement of inpatients and prediction of length-of-stay and discharge timing. We argue that there are benefits to both approaches of considering the healthcare institution as a whole as well as the patient by patient case and that ideally a combination of these would be best for improving patient flow through hospitals. We also argue that it is essential for there to be a shared dataset that will allow researchers to benchmark their algorithms on and thereby allow future researchers to build on that which has already been done. We conclude that machine learning for the improvement of patient flow is still a young field with very few papers tailor-making machine learning methods for the problem being considered. Future works should consider the need to transfer algorithms trained on a dataset to multiple hospitals and allowing for dynamic algorithms which will allow real-time decision-making to help clinical staff on the shop floor.

## 1. Introduction

When a country’s population and average age increase every year, it is inevitable that a strain is placed upon its healthcare system. This is due to the clinical attention that is generally required by older people and the increasing size of the ageing population. This is the situation faced by many countries in the world today (Andrews 2001, Tinker 2002, Oliver *et al* 2014). National media outlets can be particularly vocal about the performance of healthcare systems which makes the desire for a solution to poor efficiency in healthcare systems not only technically and economically desirable, but also politically important. The ability to cope with the demand for efficient healthcare has recently further been compromised due to the coronavirus pandemic that has swept the world which has shut down the normal operation of many healthcare institutions and reduced their capacity to treat patients significantly in many cases (Chen *et al* 2020, Hick *et al* 2020, Janbabai *et al* 2020). This has consequently increased the pressure placed on healthcare institutions as well as extending the waiting times faced by patients (Propper *et al* 2020). Despite numerous attempts by governments and hospitals to apply traditional management techniques and lean practices to improve the throughput of patients through hospitals, very little has proven effective in the long-term running of the hospital (Hall 2013, Rutman *et al* 2015). Even fewer techniques developed have proved easily extendable to multiple hospitals as a simple solution to maximising flow throughput.

It is common today for hospitals today to have digital systems in which all patient data is recorded. These are called the electronic health records and store information on the patients passing through the hospital as well as the state of the hospital at a given time. With the abundance of this data, it has become increasingly feasible to adopt algorithmic approaches to the running of hospitals. As a result, many researchers have turned to utilising machine learning amongst other algorithmic approaches in order to tackle the issue of maximising patient flow through hospitals. In using this algorithmic approach, researchers hope to create solutions which can extend to any hospital which has an electronic health record system, thereby making their solutions ‘generalisable’ to the rest of the industry. In this review we aim to provide an understanding of the landscape of research that has been developed in the field of machine learning applied to the patient flow problem.

### 1.1. What is patient flow?

Patient Flow is a term used within healthcare services to refer to the way in which patients are moved through a healthcare facility. It involves the medical care, resources, and internal systems needed to get patients from admission to discharge while maintaining a standard of quality of care and satisfaction for the patient (Hall 2013). Many works have shown that patient flow can be predictable using machine learning techniques. These works aim to use these predictions to improve the flow of patients and resources in order to provide a faster and better service to patients.

## 2. Motivation

Patient flow is a topic that has been studied extensively by various researchers of differing backgrounds. As a result the literature associated with the improvement of patient flow is vast and a diverse range of techniques from different disciplines are employed in an attempt to tackle the problem. In this review, we will primarily focus on the history of how patient flow has been handled, as well as techniques that involve the use of machine learning methods. This is, however, by no means an exhaustive review of all methods used for the improvement of patient flow. It should also be noted that this review is not intended to summarise the machine learning methods that have been applied to patient flow or the best performing models for each task (as seen in Chen *et al* 2020) and so the performances of the models will not be included. Rather, it is to provide some structure to the field of machine learning applied to patient flow, to allow researchers to see how machine learning has already been applied to the patient flow problem and where there are (to the best of our knowledge) gaps in the literature.

While some authors have attempted to tackle patient flow as a single system through a hospital, most researchers break the problem into smaller constituent problems to tackle. These constituent parts are usually associated with the key flow bottlenecks in hospitals and these are: (a) prediction of patient admissions and demand on emergency departments (EDs), (b) prediction of flow through the emergency-to-inpatient interface (i.e. handover from ED to the hospital), (c) prediction of movement of patients (and associated resource) within the hospital and (d) prediction of length-of-stay. In this review we will discuss the work published in all of these topics and how they have been used to improve patient flow through hospitals.

## 3. Outline

**Figure 1.**
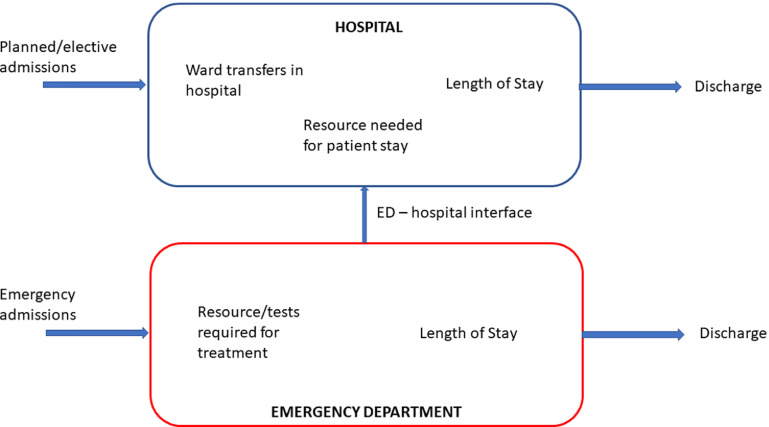
A visualisation of the process of hospitalisation and the main considerations at each stage from a patient flow perspective.

Figure 1 shows the process of hospitalisation for many hospitals with an ED (although many hospitals may also receive patients from different EDs). Hospital visits can be decomposed into two overarching types of admission: elective (planned) and emergency (unplanned). It is generally the unplanned emergency admissions which cause the greatest disruptions to patient flow through hospitals (Tancrez *et al* 2009).

Elective admissions are planned prior to their admission. As a result, the resource for these patients has been planned and there is bed space should it be needed. Elective patients have also been shown to have consistent lengths of stay in hospitals meaning they cause minimal disruption to the flow of the hospital (Kelly *et al* 2012).

Due to each emergency case being different there can be no estimate of the resource required or how long each patient will stay in hospital prior to their arrival. These therefore have become popular topics for the use of machine learning for prediction. Should these patients need hospitalisation, there is again little warning and so adapting the planning of the hospital becomes difficult.

In the following sections we will look at the work that has been carried out in applying machine learning to all of these sections of the hospitalisation process, the techniques that have been used and where we believe researchers should focus their attention on in the future to further improve patient flow.

## 4. How patient flow is currently managed

The effect of poor resource management on patient flow within the hospital is well known. Conceptually, high patient flow can be achieved by the effective balance of supply and demand within the system. If the supply of beds, staff and equipment is readily available to meet the needs of patients arriving at the door, then few perceivable barriers exist to prevent their immediate usage. However, studies of waiting lists have long shown that increasing supply in fact leads to a proportional stimulation of demand, highlighting the inadequacies of using relative need for services solely for the basis of resource provision (Feldstein and Severson 1964). If increasing supply cannot satiate demand, the optimisation of existing resources is an obvious and necessary strategy. Oredsson *et al* (2011) reviewed modern triage-based interventions designed to improve patient flow in emergency departments, demonstrating that the most significant improvements are observed through the use of fast-track and team triage approaches, indicating the importance of casemix as a fundamental consideration.

Current approaches to the management of patient flow in hospitals are typically driven by the need to report and improve upon key performance objectives. Within the United Kingdom National Health Service (NHS), the introduction of the Patient’s Charter allowed providers greater flexibility to curate local operational policies, whilst imposing stricter performance and reporting structures across the system (NHS England 2015). By specifying the metrics required to deliver an adequate level of care, the identification and treatment of bottlenecks in the system naturally become a focus of attention. Such metrics are often objective and time-based, such as the time taken for acute arrivals to be admitted or discharged. Perhaps the most significant of these targets introduced within the NHS was that of the 4 h waiting limit for ED arrivals, stipulating the need to admit, transfer or discharge a patient within this timeframe (Stevens 2004). The most widely used approach to fulfil this target in the UK is the use of the ‘See and Treat’ framework, which encourages rapid on-arrival assessment of the patients needs by an individual clinician, and allows full autonomy to that clinician to decide the treatments, referrals and investigations necessary to facilitate their care, or be discharged as appropriate. Saint Lamont (2005) discussed the benefits and limitations of this approach, including the barriers to adoption observed when additional resources or suitably trained staff are unavailable.

Anecdotally, a lack of efficiency and poor patient flow is typically perceived to correlate with a reduction in staff availability. This observation is particularly valid where patient satisfaction is concerned. A study by Thompson *et al* (1996) showed positive overall satisfaction was associated with the perception of short waiting times and accurate information delivery, rather than actual waiting times. Whilst increasing staff within the emergency department may improve turnaround times for rapid triage and discharge of non-urgent cases, it is less likely to result in an improvement for patients requiring admission, as shown by Bucheli and Martina (2004), indicating that the true bottlenecks exist further in the pathway beyond the emergency department. This fact has been clearly recognised in recent guidance, where the focus on enabling patient flow has shifted away from the performance of the ED and towards acute networks and support services (Ham 2017). At the one end, Clinical Streaming has been introduced as the process by which patients are assigned to one of several parallel pathways, according to their care requirements, allowing for more structured and reliable coordination of support services within the hospital. At the other, Discharge to Assess (D2A) models emphasise the need to address unnecessary delays in discharging clinically optimised patients from hospital, due to a lack of funding or support within the community (Hyslop 2020).

## 5. Machine learning for patient admissions

### 5.1. Prediction of emergency admissions

The number of patient admissions to the hospital is arguably one of the most important aspects of patient flow. This determines the demand that is placed upon the hospital and therefore affects how patients can be treated. The importance of predicting patient admissions is reflected by the number of publications in this area. However, with little information on patients prior to their arrival it is also one of the most difficult areas of patient flow to create accurate predictions.

Boyle *et al* (2008, 2012) and Batal *et al* (2001) predict the number of emergency admissions using multiple regression. They frame the problem such that they forecast for daily admissions as well as weekly and monthly admissions. The use of regression is for interpretability of the predictions as well as the development of a simple model to improve the chance of being able to generalise to other hospitals. As mentioned previously, due to limited information on these patients prior to arrival, the authors use the days of the week and national holidays as features.

Whereas the aforementioned studies approach the problem as a static prediction (i.e. using information from a snapshot in time to make predictions), Tandberg and Qualls (1994), Au-Yeung *et al* (2009), and Schweigler *et al* (2009) treat the problem as a time-series. They use autoregressive models to account for the trajectory of the numbers of patients. This approach is more likely to be successful than a static approach due to the incorporation of data close to the event of interest. However, the benefit of a static approach (if the model is accurate) is that a prediction can be made at an early stage and action can be taken based on that prediction without needing to wait for the time-series to unfold. These time-series approaches also perform regressions to predict patient volumes in the coming days, weeks and months.

While the seasonal features such as weather and time of the year have been shown to be helpful with predicting patient numbers, they are not patient-specific and therefore are limited in their use for predicting when a patient will be admitted to hospital. As a result, LaMantia *et al* (2010) and Artetxe *et al* (2020) consider predicting patient readmissions to the ED instead of predicting any given admission. In doing so they are able to utilise the wealth of data already recorded by the hospitals on individual patients and identify markers that indicate high risk of readmission in an emergency. Hosseinzadeh *et al* (2013) use Naive Bayes and a decision tree in order to classify patients who are going to be readmitted to hospital using their health records as features. These works generally pose the readmission problem as predicting readmission within the next 30 d as this has the most impact on the health and welfare of the patient, as well as the scheduling of the hospital (Leppin *et al* 2014).

A problem that can arise due to these readmission predictions is that patients can be readmitted for various issues (for example a patient who was hospitalised for cardiac issues might need rehospitalisation for breaking their leg). Considering this type of readmission is not very useful for the hospital or the patient, as it is not indicative of an underlying condition and so the health records of the patient will not be useful for this prediction. To get around this issue, many authors have conditioned their prediction of admission on subsets of patients with certain underlying conditions. Shameer *et al* (2017) use a naive Bayes classifier to predict readmission and only considers a subset of patients with heart failure. They only consider a readmission to be valid if the patients are readmitted with heart failure within 30 d. Kalagara *et al* (2018) also condition their problem on a subset of patients who have had a neurosurgical procedure carried out and compare the performance of their model (trained used gradient boosted trees) using features available during the patients stay versus features that were obtained after the patients discharge. Naturally the model with access to features after the patient discharge performed better, however it is very difficult in most situations to obtain features post-discharge. Min *et al* (2019) carry out a similar study but consider patients suffering from COPD. They investigate various machine learning methods and find that gradient boosted trees offer the best prediction of readmission accuracy for their dataset. They also utilise recurrent neural networks in order to treat the problem as a time-series problem but the performance is significantly worse. In fact there are very few works that treat the prediction of readmission as a time-series due to the difficulty of obtaining data on patients post-discharge (Arora *et al* 2010).

### 5.2. Scheduling instead of admissions

The ultimate aim of all of the works mentioned in section 5.1 is to provide the hospital with an understanding of the volumes of patients that may be attending the ED. By forecasting this (and if the model is accurate) the hospitals may then plan the appropriate resource (including staff, tests and making equipment available) in order to be able to cope with the demand placed on them. For low numbers forecast, hospitals may also then reduce the required resource that is on standby which can lead to cost savings (Thungjaroenkul *et al* 2007).

Some authors however approach the problem from the scheduling perspective. This is different in that whereas predicting admissions makes the assumption that resource can be altered to meet demand, the scheduling approach does not. With this approach authors assume that there is fixed resource and how it is used can be optimised with varying patient numbers.

Rosemarin *et al* (2019) define the ED scheduling problem as needing to satisfy the following constraints: the schedule must minimise the risk of adverse consequences, minimise patient waiting time, minimise patient length-of-stay, minimise ED crowding and minimise interruption to caregivers. They use a mixture of health record data of the patients and data on the status the ED to reconstruct the state of the ED when the patients were there. They then use a mixed integer linear program to optimise these scenarios, maximising throughput while being constrained by the aforementioned constraints. They then train a deep learning architecture on this optimised data and use it as a ranking system to predict the optimal patient-caregiver pair in the ED.

Some authors prefer to allow the machine learning algorithms to discover the optimal policies instead of optimising the problem themselves to learn from. This is seen in Lee and Lee (2020a) where a deep Q network (a reinforcement learning algorithm) is used to learn the optimal policy of treating patients in the ED. In order to do this a simulation is made of the ED which will allow the agent to take exploratory moves essential for reinforcement learning. The state of the model is defined as the distribution of acuity (sum of patients at each acuity level) within the ED as well as the distribution of needed treatment type. The action of the agent is to rank the next patient that needs to be seen meaning it is also a patient priority-ranking system. Krämer *et al* (2019) also present a priority ranking system based on severity prediction, but go as far as predicting whether patient presentations to the ED should be treated as elective visits given their low severity. They do this using the primary diagnosis code of the patient, however there may be difficulties in expanding this tool to other hospitals given that many hospitals assign diagnosis codes after the patient is discharged from hospital and not at admission.

Yeh and Lin (2007) and Arisha and Abo-Hamad (2013) approach the scheduling problem slightly differently in that instead of ranking the priority patients in the ED, they instead aim to design the staffing schedules. They do this using genetic algorithms and allowing the staffing schedules to be updated and ‘evolve’ to a point where they are suitable for the demand placed on the ED. This approach makes the assumption that should the staffing level be predicted accurately, then there will be no need to prioritise patients in the ED as there will be enough staff (and resource) to process them.

**Figure 2.**
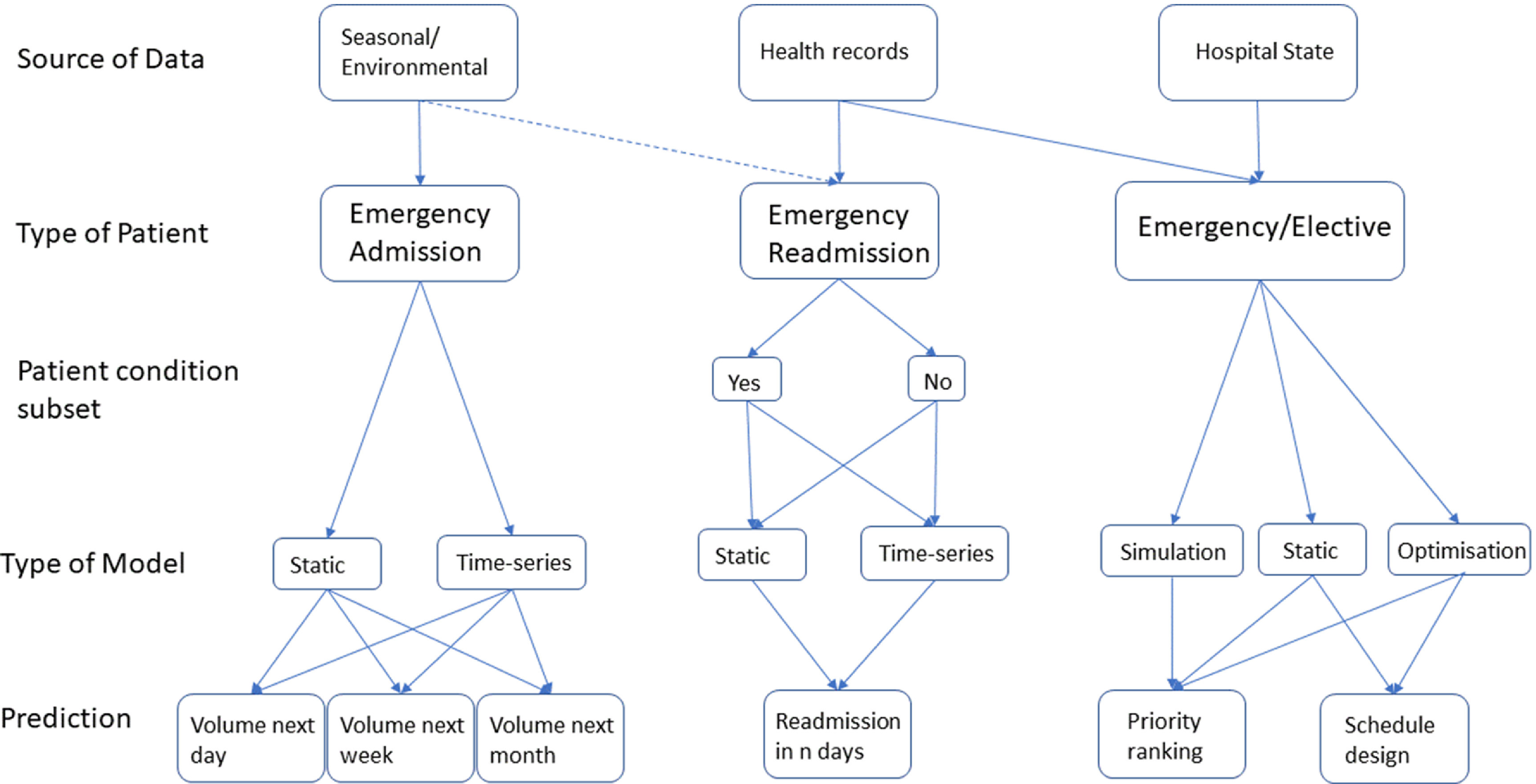
Visualisation of the studies that have been carried out regarding using machine learning to predict admissions and scheduling in the ED. Dashed lines indicate some studies opt to use these features.

Figure 2 shows the works that have been conducted so far on the prediction of admissions and scheduling in the ED. This is by no means an exhaustive summary but we aim to provide some structure to help other researchers understand what work has been conducted in the field of machine learning for patient flow through the ED. Table 1 further outlines the problems that have readily available datasets for prediction, and what models are popularly used to tackle the prediction problem in the literature. A lack of a readily available dataset for priority ranking is due to priority generally not being recorded in hospital EHRs. Readily available in this instance refers to existence in a typical hospital database and not that it is easily and openly accessible.

**Table 1.** Popularity of different methods and data availability for each of these problems.

	ED-admission problem			
	Volume pred.	Readmission pred.	Priority rank	Schedule
Labelled datatset readily available?	$\checkmark$	$\checkmark$	✗	$\checkmark$
Regression methods popular?	$\checkmark$	$\checkmark$	$\checkmark$	✗
Classification methods popular?	$\checkmark$	$\checkmark$	$\checkmark$	$\checkmark$
Genetic methods popular?	✗	✗	✗	$\checkmark$

### 5.3. Machine learning in elective admissions

We have primarily focused on machine learning applied to emergency admissions as this is the larger body of research in the field. The stochastic nature of these admissions in terms of number and type of admission means that these are the most disruptive to patient flow in a hospital. Elective patients are generally planned for and so resource is available to treat them.

There are however studies that also apply machine learning to the admission of elective patients. In the study conducted by Nelson *et al* (2019), the authors use machine learning to assess whether or not patients will actually attend their scheduled appointments in hospital. Despite the resource being prepared for these patients, a no-show will result in a waste of this resource and this work aims to provide a way to then re-direct that resource. The authors use information on the history of the patient with a gradient boosting machine to get a strong predictive accuracy. Srinivas and Ravindran (2018) carry out this same prediction of no-shows to elective appointments, however they then leverage the risk of no-show in order to update the scheduling system of the hospital.

With many health systems providing long waiting times for appointments (Xavier 2003, Dimakou 2013), another important factor when it comes to elective patients is prioritising patients in the schedule. Yousefi *et al* (2019) approach this by first using a clustering algorithm to group patients into different priority categories. They then treat the schedule as a Markov decision process where waiting time for patients in the high priority clusters is to be minimised.

These approaches can be difficult to validate due to their direct impact on the scheduling of appointments. As a result, there is no chance to verify if the patients turn up or not once the schedule is changed. They also rely on historical behavioural data (such as how many times a patient has missed an appointment before) which are not stationary distributions and therefore limit how successful supervised learning can be in this domain in the long term.

### 5.4. Summary

Overall, the application of machine learning to predicting emergency patient admissions and scheduling is well-explored. Works are generally split between emergency and elective patients with further subdivisions according to the data used, the models used and what is being predicted (see figure 2). Very few works validate their models in hospitals in real-time, most using a retrospective test-set to assess performance. Furthermore, some models are difficult to validate due to being designed to intervene in the admission and scheduling process.

There is also very little connecting these studies. Most work is carried out with the data from the hospital that the authors are associated with and built around that. Due to hospitals being different, that leaves little scope for building on previous work or developing models that can be used universally. A public dataset that could be held as the gold-standard for patient flow would aid in this significantly as a benchmark for experiments.

## 6. The emergency-inpatient interface

The emergency-inpatient interface is an ill-defined area of many hospitals (Staib *et al* 2017). There is usually a lack of clarity on the ownership of this space of the hospital and who should manage the handover of patients from the ED to an inpatient setting. As a result of this lack of clarity, it should come as no surprise that there is much published on making predictions across this gap in the hospital. While it may seem like an obvious task to predict which patients need admission to hospital from the ED, it has been shown that this is not a trivial task (Beardsell and Robinson 2011). Whereas the works discussed in section 5 aim to provide predictions for planning (such as expected numbers or schedule planning), the predictions of the works found in this section are primarily designed for decision-support.

A natural question that can be asked is if admission to the hospital from the ED can be predicted. Hong *et al* (2018) and Graham *et al* (2018) show this can be done using multiple machine learning models including a logistic regression, XGBoost and a deep fully-connected neural network. They show this is possible using historic patient information as well as information from triage. This does however limit the potential use to patients who already have electronic health records. Leegon *et al* (2005) and Raita *et al* (2019) therefore also carried out this prediction but only using a few variables that are measured early in the ED admission process and showed using a Bayesian network that admission to hospital can still be accurately predicted. Sun *et al* (2011) echo this sentiment, setting up their classification such that the clinical staff may predict the risk of whether an inpatient bed is needed or not as soon as triage is complete in the ED. This prediction is then further augmented with the inclusion of using the free-text written by the triage clinical staff as features to improve the performance of the model (Zhang *et al* 2017, Sterling *et al* 2019).

As was the case for prediction of admissions in section 5, many authors find it useful to consider certain demographics of patients. An example is in Lucke *et al* (2018) where a multiple logistic regression is used to predict hospital admission from the ED for a cohort of patients over 70 years old and another below. This is due to older patients generally being more at risk of admission and so by creating a model conditioned on age, they are able to better predict those most at risk of admission. In Mowbray *et al* (2020), elderly patients are considered to be those aged 75 and over, however they also show that accurate predictions of admission can be made for an elderly cohort of patients.

Another demographic that is often targeted for prediction is that of paediatric patients (Walsh *et al* 2004, Marlais *et al* 2011). In these studies, logistic regressions are used to predict whether a paediatric subset of patients will require admission to the hospital. Once again, by creating a separate cohort for these patients, they can make predictions comparing patients to other similar patients, rather than comparing with older patients who have different physiologies. This introduces a trade-off of improving model accuracy while reducing how generally the model can be applied.

Further subsets of paediatric patients have been made for example by considering those patients suffering from asthma exacerbation and predicting those most likely to be admitted to hospital for treatment (Patel *et al* 2018).

To augment the performance of a model predicting paediatric admissions to hospital from the ED, the textual data recorded during triage can also be used as features (Roquette *et al* 2020). Natural language processing techniques have been used in order to extract useful information which has been shown to improve predictability of admission.

### 6.1. Predicting inpatient resource utilisation

Many of the studies that are created in predicting admission to hospital focus on subsets of patients with certain conditions. As these patients will require the same treatments and specialist staff to treat them, this can be seen as resource prediction for patients being admitted to the hospital from the ED.

An example is in Ong *et al* (2012) where heart-rate variability in the ED is used alongside other demographic information on the patient as input features to a support vector machine. This is then used to create a score on the likeliness of cardiac arrest occurring in the next 72 h. While this is not strictly framed for patient flow, this prediction allows clinicians to plan for resource in the cardiac department. Predicting whether or not a patient is septic is also important for patient flow in terms of resource planning. As a result, models predicting whether or not ED patients are suffering from sepsis have been developed (Horng *et al* 2017, McCoy *et al* 2017, Delahanty *et al* 2019). The authors use a mixture of information available at triage, demographic information and free-text to make prediction of whether or not the patient is septic, which if accurate, could allow planning of their treatment before the patient becomes critically ill.

In fact, there have been many such studies predicting whether or not a patient is suffering with a certain condition in the ED which allows resource planning. These include predicting if a patient is suffering from acute kidney injury (Martinez *et al* 2020), requires intensive care (Fernandes *et al* 2020, Finkelstein 2020), is suffering from a urinary tract infection (Taylor *et al* 2018), have bacterial infections (Ramgopal *et al* 2020) as well as predicting emergency hospitalisation of patients undergoing chemoradiation (Hong *et al* 2018).

While these predictions are useful for planning patient flow, they are not explicit predictions of admission. A more explicit approach is seen in Luo *et al* (2019) where the classifier is trained to predict admission to hospital of patients suffering from bronchiolitis.

While predicting admission to hospital from the ED is useful, a greater level of granularity, such as which departments in the hospital the patient will be admitted to, is more useful to clinical staff. An example is seen in Lee *et al* (2020) where rather than predicting admission, they predict the disposition of the admitted patient, choosing out of intensive care units, telemetry units, general practice units and observation units. As these ‘ward types’ tend to have separate resource, they are better able to adapt their resource according to the predictions made. This approach is also seen in El-Bouri *et al* (2020) where the authors also classify into ‘ward types’ to provide a similar level of granularity to the hospital admission prediction problem. However, in this case they use medical, cardiac, neuro, trauma, intensive care, surgical and general/obstetrics and gynaecology as their ward groupings. They develop a novel ‘interpretable’ layer for their deep neural network to guide information collection at triage and train the model using curriculum learning. El-Bouri *et al* (2020) further augment their model by using reinforcement learning to allow an agent to carry out the curriculum learning that maximises the performance of predicting where in the hospital patients will be admitted to. In order to make as general a model as possible, these studies of patient disposition do not consider subsets of patients but rather the entire population of the ED to replicate daily working conditions.

**Figure 3.**
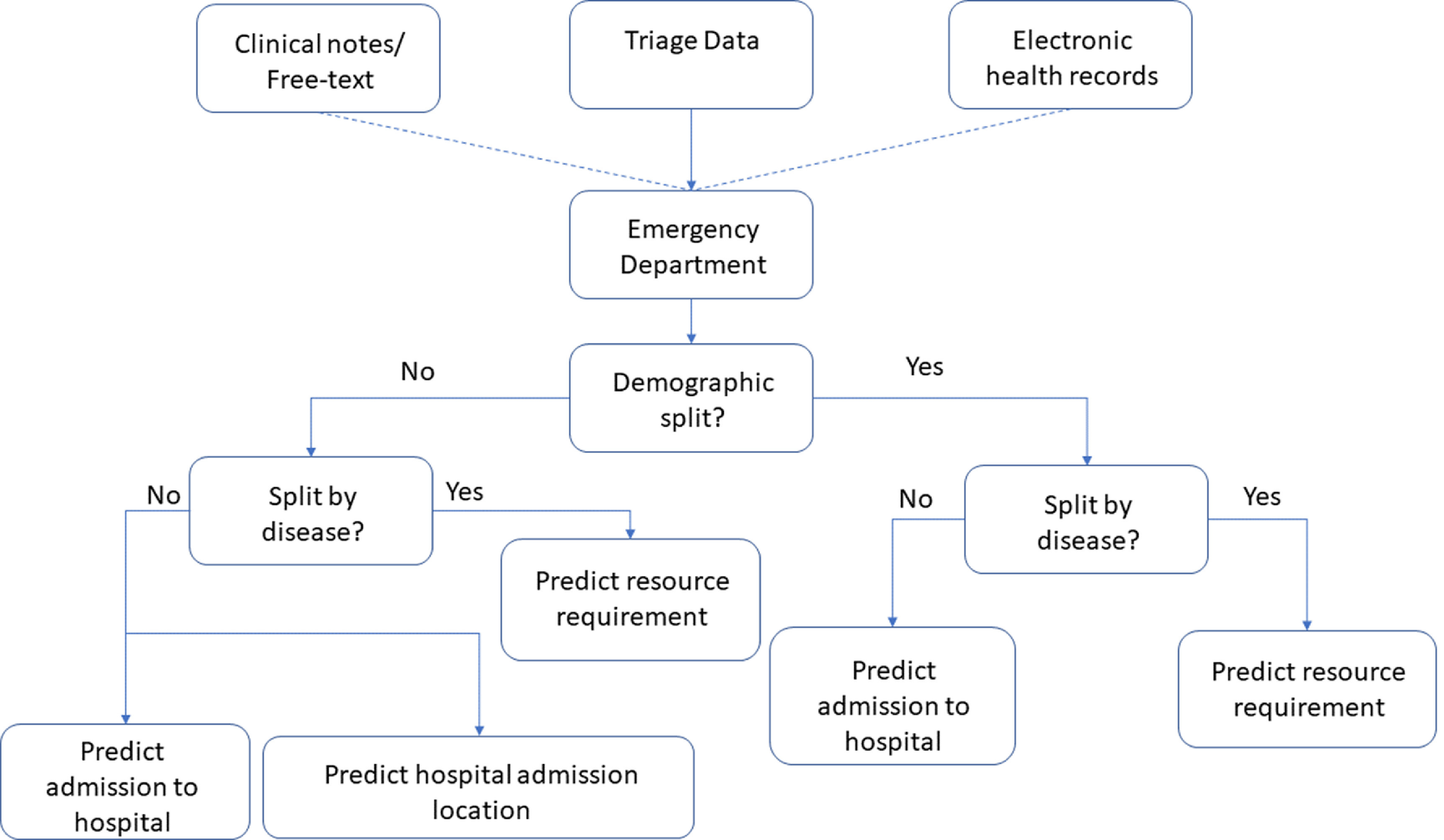
A decision tree showing how the studies that have been conducted on predicting movement from the ED to hospital are structured. Dashed lines indicate that these features are used in some works but not all.

Figure 3 shows the general structure of works that have been conducted on predicting flow from the ED to hospital. It should be noted that as all of these works consider flow from the ED, all patients considered are emergency patients. Table 2 shows how readily available labelled datasets are for the EDii prediction problems and the popular approaches to tackling them. It should be noted that readily available here means data that would generally be saved on a hospital EHR and not data that would be easily accessible on a public dataset.

**Table 2.** Popularity of different methods and data availability for each of these problems.

	EDii problem		
	Hosp. admission	Hosp. admission loc.	Resource req’ment
Labelled datatset readily available?	$\checkmark$	$\checkmark$	$\checkmark$
Regression methods popular?	✗	✗	✗
Classification methods popular?	$\checkmark$	$\checkmark$	$\checkmark$
Bayesian methods popular?	$\checkmark$	✗	✗

### 6.2. Summary

We have seen in this section that the emergency-inpatient interface in hospitals, while being ill-defined in practice, is well researched using machine learning. Authors predict admission from the ED in order to provide information for clinical staff to prepare space should it be needed. To improve the performance of the classifiers, many authors condition their models on the demographics of the patients (e.g. elderly or young patients) or on the patient disease (patients suffering from the same ailment in the ED). In order to provide greater granularity on which resource will be used in the hospital, some authors also predict which ‘ward type’ will be used by the patient to be admitted to the hospital.

However, once again there is little connecting these studies. None of the studies reviewed build off each other or use the same dataset for comparison. Furthermore, the definitions used to categorise patients vary by paper. As was seen when categorising elderly patients some studies use 70 and over and some use 75 and over. Clearly it would be beneficial to have an agreed range to make models more comparable. This further emphasises the need for a shared, publicly available dataset for use when creating machine learning models for patient flow. All definitions of demographics should be included in the dataset so that researchers make valid comparisons to models. It will also be beneficial in allowing researchers to compare their methodologies and validate them on the same dataset as others as well as apply them to their own hospital’s data. This will also make research more consistent, allowing researchers to build and improve upon each others models instead of applying similar models to similar problems using different data.

## 7. Intra-hospital resource management

Once patients have been admitted to hospital, there is yet another layer of resource flows that need to be considered. Patients can be transferred between wards, need tests carried out and must be moved to use certain equipment such as MRI scanners. These all require staff to carry out the movements and therefore place a demand on the resource of the hospital. As this resource is part of that needed to deliver the patients through hospital to discharge, it is relevant to patient flow.

### 7.1. Ward transfer

The most common way in which machine learning is used to provide predictions for ‘inpatient flow’ is through predicting if patients will be transferred to another ward. Note that while in section 6.1 we considered studies which investigated patient degradation as a signal for resource preparation, we will not consider degradation for inpatients as a signal for resource prediction. This is due to hospitalised patients generally being admitted to wards that are capable of handling patients in their condition. It is also due to the fact that using machine learning for the monitoring of inpatients for degradation has a very rich literature and would require a review of it is own (Clifton *et al* 2015). As a result, we only focus on works that explicitly predict admissions or transferrals of patients.

#### 7.1.1. ICU transfer

By far the most popular type of prediction to make in the inpatient setting is predicting admission of a patient to the ICU. This is due to the fact that the ICU is a resource intensive area of the hospital and any way of informing the planning of this unit is beneficial to the running of the hospital (Skowronski 2001).

Wellner *et al* (2017) use a logistic regression to show that it can be predicted that a patient will need admission to the ICU 16 h ahead of time. Furthermore they demonstrate this using data from three separate institutions, helping validate their model. Desautels *et al* (2017) carry out the same investigation in a tertiary care hospital but consider readmissions to the ICU in 48 h. This is also explored by Yoon *et al* (2016) who develop a ‘Bayesian belief system’ to predict admission to the ICU, but this time 9 h before it is requested by the clinician in charge. An NLP approach has also been investigated in Khattak *et al* (2019) where the online messages of doctors and nurses to each other are used in order to predict transferral of a patient to ICU 3 d prior to the event taking place. It should be noted that for all of these studies, the outcome being predicted is different and so the studies cannot be compared.

Echoing the narrative presented in section 5, many researchers have also considered predicting readmissions of inpatients to the ICU. This is seen in Rojas *et al* (2018) where the authors investigate which patients, who were previously in the ICU, will be readmitted from their inpatient ward. To predict this they use a gradient boosting machine with features derived from the electronic health record of the patient as well as various blood tests that were taken. A time-series approach to this prediction was investigated by Lin *et al* (2019) where an LSTM was used and trained on the ICD-9 embeddings of the patients who had previously been admitted to the ICU, their demographics and the chart event features of the patients. They show a strong prediction accuracy when considering if a patient will be readmitted to the ICU within 30 d of their discharge.

Once again, conditioning the dataset on the demographic in question is utilised for the inpatient setting. Rubin *et al* (2018) demonstrate using adaptive and gradient-tree boosting that they can predict the transfer of a child to the paediatric ICU 8 h preceding the transfer. The prediction of transfer to paediatric ICU is also carried out in Zhai *et al* (2014) where a logistic regression is used to predict their transfer within the first 24 h of their inpatient status.

We again see works where the datasets (and therefore the models) are conditioned on the co-morbidities of the patients. Lee *et al* (2019) condition their dataset on patients who have undergone cardiac surgery and predict whether these patients will be readmitted to the ICU. They use a logistic regression with L1 regularisation to provide interpretability to their model, but also use a causal inference method to compare their findings. They find that there is little agreement between the two methods of feature importance ranking.

### 7.2. Resource management

During a patient’s stay in hospital, various tests may be requested to help clinicians gain a better understanding of the patient’s condition. These tests are also an important part of the patient flow process and timely testing helps to improve flow through the hospital. An example is seen in Molaei *et al* (2016) where the authors investigate whether or not they can predict if inpatients with traumatic brain injury require a CT scan using ‘cost sensitive’ random forests. In doing so, they aim to create a prioritisation system for scanning, which will allow faster treatment of patients and therefore a better patient throughput.

Another way in which resource management has been tackled with machine learning is in the scheduling of laboratory samples that need to be processed (Williams *et al* 2019). Again, by scheduling these samples in an efficient way, this allows patients to be treated more quickly in the hospital, and in some cases prevents the unnecessary hospitalisation of a patient.

These examples can be seen as assessing the risk of resource utilisation on a patient-by-patient basis. A more high-level view is used in Vieira and Hollmén (2016) where all resource is pooled together (anything including staff or use of machinery). Random forests are used to perform regression on the expected resource use in the next 30 d. While this has limited use to clinical staff due to the lack of granularity, it may be useful for budgeting purposes.

### 7.3. Hospital-wide flow

There are very few works that seek to predict the full patient journey through a hospital using machine learning. This may be due to the fact that transfers of inpatients is generally quite rare due to most inpatients being admitted to a ward that is capable of providing the appropriate care for them. Xu *et al* (2017) treat the hospital journey as a point process. They use a generalised linear model to predict the next location a patient will be transferred to as well as the dwell-time in that unit. They utilise the MIMIC-III dataset (Johnson *et al* 2016), which is an ICU based dataset and so the transitions they predict are between various types of intensive care unit. However, in terms of predicting the inpatient journey, this is a promising direction. Expanding to the entire hospital, it is possible to predict movement of patients between wards as well as for the use of machinery. Also predicting the dwell-time will allow for better planning of the flow of patients.

### 7.4. Summary

Of the four parts of the hospitalisation process that we have defined, the inpatient setting is the one in which machine learning has been used the least. The majority of studies investigate the transferral of inpatients to the ICU due to the resource-intense nature of ICUs. There have also been limited attempts at utilising machine learning to predict the expected resource that will be required by a hospital, either as a whole, or on a patient-by-patient basis. Very few works again have attempted to predict the whole hospital journey using machine learning. A common inconsistency throughout the literature is the prediction lookahead time that is considered. Standardising the lookahead time will allow studies to be more comparable and again, crucially, build upon previous work to further improve and integrate the field.

**Figure 4.**
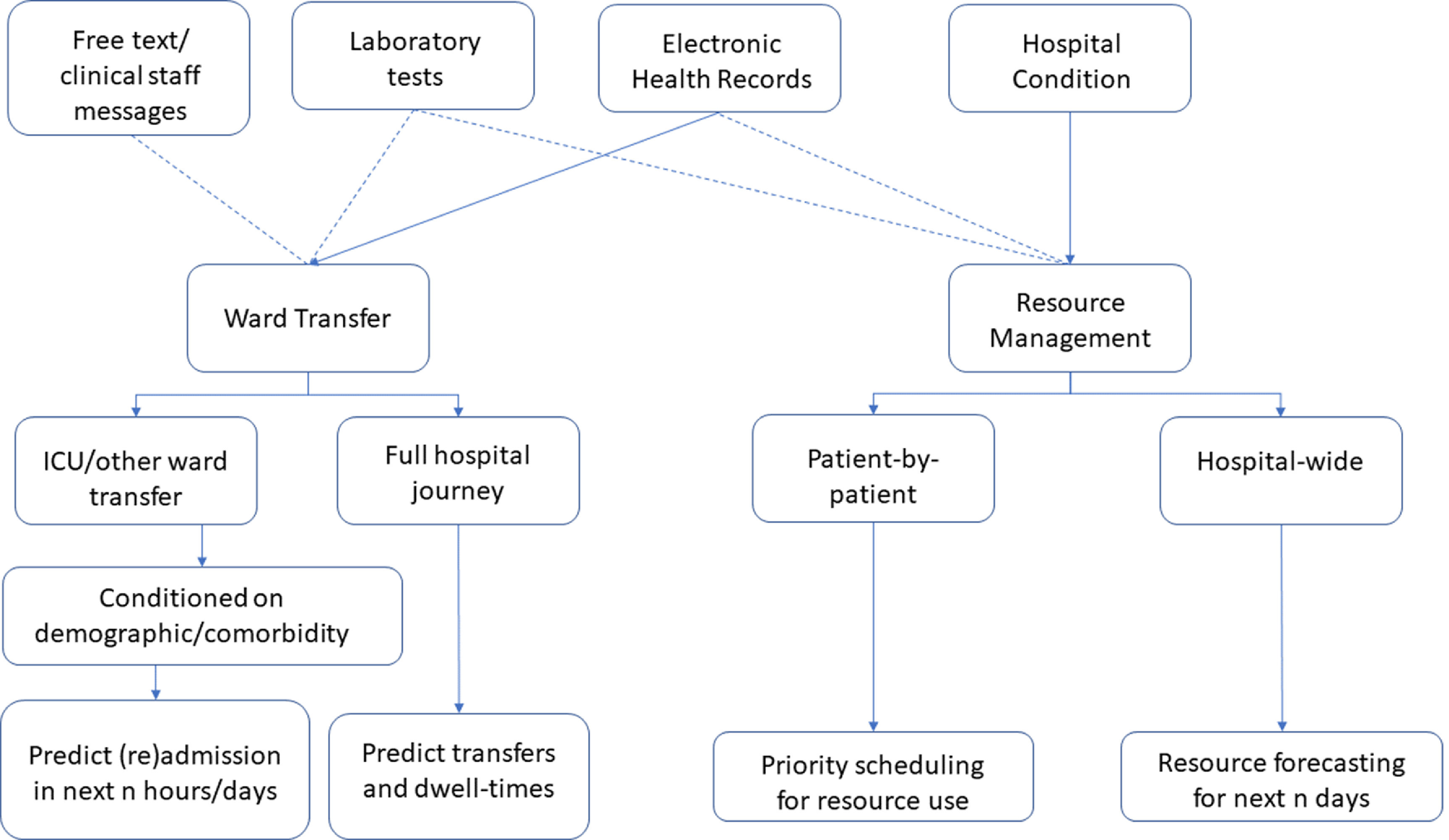
Visualisation of the studies carried out on using machine learning to aid in the inpatient journey. Dashed lines indicate some studies opt to use these features.

As the vast majority of studies are conducted with a clinical need in mind, this may reflect that the inpatient journey is not seen as a very important part of the patient flow problem. Figure 4 shows the structure of the studies that have been carried out in this area of patient flow. Table 3 shows the data availability for these prediction problems and popular methods used to tackle them.

**Table 3.** Popularity of different methods and data availability for each of these problems.

	Intra-hospital prediction problem		
	Ward (re)admission	Transfers	Resource forecast
Labelled datatset readily available?	$\checkmark$	$\checkmark$	$\checkmark$
Regression methods popular?	$\checkmark$	✗	$\checkmark$
Classification methods popular?	✗	$\checkmark$	$\checkmark$
Point processes popular?	✗	$\checkmark$	✗
Bayesian methods popular?	$\checkmark$	✗	✗

## 8. Discharge prediction

The importance of discharging patients in a timely fashion for patient flow cannot be over-stated. Long patient stays incur greater cost to the healthcare institution and reduce capacity for new patients to be admitted (Rotter *et al* 2008, 2010). As a result, a standard metric of the quality of care being provided is the patient length-of-stay (LOS) (Brasel *et al* 2007). Patients who are admitted for long periods of time (either due to condition or due to having no appropriate discharge destination) are commonly referred to as ‘bed-blockers’ and can constitute a significant proportion of the hospital population (Coid and Crome 1986, Styrborn and Thorslund 1993, Mustafee *et al* 2012). Early recognition of the patients likely to have a long LOS should therefore allow for the planning of their treatment by the hospital, such as their admission to long-stay wards and beginning preparations for their discharge.

It should therefore be unsurprising that many researchers have seeked to employ machine learning in order to predict the LOS of patients in order to provide hospitals with a better idea of how much resource will be required for patient stays. Note that the prediction of LOS or of discharge are essentially the same as they both aim to predict when a patient is able to leave the hospital. We will refer to both types of prediction simply as ‘discharge prediction’.

Discharge prediction can be separated into two separate subcategories for emergency and inpatient settings. In the emergency context, predicting the LOS of patients helps to understand whether the ED is at risk of overcrowding or not. In the inpatient setting, predicting the LOS is useful for the planning of patient admissions and preparation of post-discharge care should it be needed.

### 8.1. Discharge in the emergency department

Discharge from ED has been treated as a classification as seen in Rahman *et al* (2020). The authors predict if a patient will be in the ED for longer than 4 h or not. They use features that are available early in the ED process to train a decision tree binary classifier. This approach is mimicked in Sariyer *et al* (2019) where various learning algorithms are experimented with to classify patients according to their length of stay in the ED. Azari *et al* (2015) acknowledge the large imbalance there tends to be in LOS datasets (with far fewer patients having long LOS), and present an ensemble method combined with multiple logistic regression to overcome this imbalance. However in this work they define a long stay as patients in the ED for longer than 14 h.

Rather than classify patients according to their likely LOS category, some authors prefer to use regression to predict each patient’s LOS in the ED. Combes *et al* (2014) use linear regression model to predict the likely LOS of each patient presented to the ED. Ding *et al* (2010) instead use quantile regression but once again for the prediction of LOS in the ED. Feedforward neural networks have also been used for regressing the likely LOS of patients (Gül and Güneri 2015). One advantage to this approach of regressing the probable LOS is that there are no longer inconsistencies between studies on what is defined as a long-stay. However, this approach is also more difficult to train and achieve an accurate model in practice.

### 8.2. The inpatient setting

Predicting the LOS of patients in the inpatient setting is significantly more popular as a research area than in the emergency setting. This may be due to a prediction of LOS in the ED being less actionable than in the hospital where preparations can be made to ready a patient for discharge.

A hospital-wide approach is adopted in Pendharkar and Khurana (2014) where a regression tree is used to predict the LOS of patients admitted to hospitals in Pennsylvania using data that is available at the time of admission. This approach is also applied in Tanuja *et al* (2011), this time using a feedforward neural network to regress the LOS. These predictions are carried out at the time of admission. An alternative approach is to implement a classifier every day before discharge and predict the patients who can be prioritised for discharge as seen in Barnes *et al* (2016). In framing the problem in this way the authors exploit a static model for a dynamic problem by repeatedly applying the algorithm prior to discharge sessions at the hospital. They use a classification decision tree to prioritise patients ready for discharge.

Predicting discharge has also been approached as a time-series problem. In McCoy *et al* (2018) an autoregressive integrated moving average model is used to incorporate a time-series of seasonal data to predict hospital discharge volume. They compare this with using Prophet (Taylor and Letham 2018), an additive regression model developed by Facebook Research for forecasting seasonal trends, for the same task. An NLP approach has also been used where the clinical notes from the ED are used in order to predict if a patient will be admitted to the hospital for more than 2 d (Bacchi *et al* 2020).

As has been a common theme throughout this review, discharge predictions are also conditioned on patient demographics. In other sections this is primarily to improve predictive performance amongst patient subgroups. However, in discharge prediction this is due to certain patient subgroups being more likely to be ‘bed-blockers’ such as elderly patients (Launay *et al* 2018). To maximise clinical utility it is more effective to condition the training dataset on these subgroups and apply the algorithms to these patients only. An example is in Elbattah and Molloy (2016) where a regression forest is used to predict the LOS of elderly patients in a hospital and a random forest is used to predict the location of discharge for these elderly patients. These predictions are used in conjunction with a discrete-event simulation in order to simulate the flow through an Irish hospital. Children are also a cohort of patients in which there can be great variability in LOS. To address this, Castiñeira *et al* (2020) use a gradient boosted tree to classify whether or not a child will be a long-stay patient in the paediatric ICU (with long-stay being defined as a stay of greater than 4 d). They also use the static model for a dynamic problem approach by extracting features from the time-series of the patient’s vital signs and repeatedly feeding these to the classifier. Note that this prediction concerns the LOS within a ward and not the hospital stay as a whole.

As with conditioning on demographics, conditioning on co-morbidities is also done in discharge prediction. In fact, this tends to be the most popular form of setting the problem due to patients with different ailments and treatments generally requiring different recovery times.

One such prediction is carried out for patients with congestive heart failure in which the authors apply a static cubist model (Quinlan 1998) dynamically as data is updated during the patient stay (Turgeman *et al* 2017). The model is used to regress the likely LOS in hospital of the patient.

Further discharge predictions have been carried out on patient cohorts who have suffered from stroke (Al Taleb *et al* 2017), patients who have suffered hip-fracture (Elbattah and Molloy 2016), patients suffering from schizophrenia (Kirchebner *et al* 2020), patients admitted for cardiac care (Daghistani *et al* 2019), patients post-brain tumour surgery (Muhlestein *et al* 2019), patients who have undergone total hip-arthroplasty (Ramkumar *et al* 2019) and patients who have undergone surgery due to colorectal cancer (Stoean *et al* 2015). In all of these studies, there is no consensus for defining a ‘long-stay’ patient.

### 8.3. Summary

Discharge prediction is one of the more popular areas of patient flow for researchers to apply machine learning. Discharge prediction has been carried out by either predicting whether a patient is likely to be long-stay or by directly regressing the expected LOS of the patient. It has been applied to both emergency and inpatient settings. In the inpatient setting, studies have conditioned their datasets according to demographic. There have also been studies that condition their dataset according to the comorbidity or treatment that the patients of interest have undergone.

**Figure 5.**
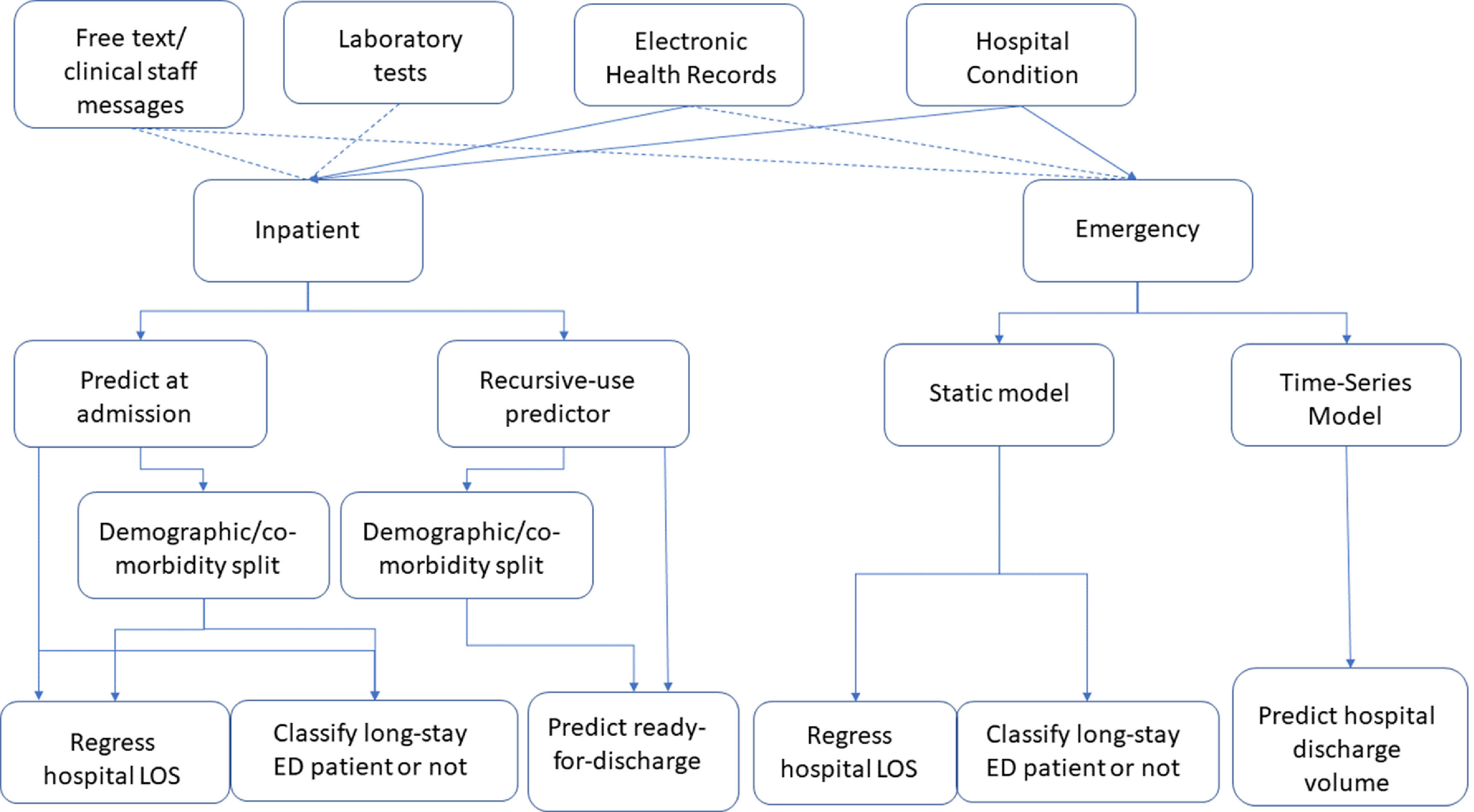
Visualisation of the studies carried out on discharge prediction. Dashed lines indicate some studies opt to use these features.

A clear inconsistency between studies is the definition of a long-stay patient. Having a common dataset with pre-defined long-stay patients will improve the ability of researchers to compare models and build upon previous work. Figure 5 shows the structure of the literature published in this field. Table 4 shows data availability and popular methods used to tackle the discharge problems. It should be noted that the difficulty with a labelled dataset for discharge readiness is that generally it is not recorded when a patient is ready for discharge but when they actually are discharged.

**Table 4.** Popularity of different methods and data availability for each of these problems.

	Discharge prediction problem		
	Hospital LOS	Long-stay prediction	Discharge ready
Labelled datatset readily available?	$\checkmark$	$\checkmark$	✗
Regression methods popular?	$\checkmark$	✗	✗
Classification methods popular?	$\checkmark$	$\checkmark$	$\checkmark$
Point processes popular?	✗	✗	$\checkmark$
Bayesian methods popular?	✗	✗	✗

## 9. The future of machine learning in patient flow

The current research efforts in the discipline of machine learning in patient flow have demonstrated the feasibility and potential of machine learning to optimise patient flow in all of the four subcategories outlined our study. However, due to the difficulty of expanding and scaling machine learning models across different healthcare contexts and institutions, the current research efforts are still removed from delivering value in the routine and daily management of patient flow in healthcare institutions. In this section, we outline the future research opportunities to advance the applicability of machine learning in patient flow.

### 9.1. Priorities in patient flow

While all of the problems outlined in the above review are important for clinical practice, solving some of these problems is more urgent than solving others. An example of a high-priority problem to solve is predicting readiness for discharge. One of the greatest problems dealt with in patient flow is the ‘bed-blocker’ phenomenon whereby patients do not have appropriate destinations to be discharged to. Predictions of readiness for discharge will not solve the lack of space in care homes, however it will allow for more effective allocation of the time and attention of clinical staff.

An equally important task is the prediction of ED admissions. This represents the front-end of the hospital with the discharge readiness representing the back-end. Being able to accurately predict patient admissions numbers in the ED would allow for accurate planning of staffing rotas thereby reducing costs and time wasted. It would also greatly improve the care provided for each individual patient.

Following on from this, should these predictions not be accurate enough, solving the ED-inpatient interface problem would be the next most important. This prediction would prevent the filling up of the ED due to inability to transfer patients into the hospital. Having an accurate model here would create a more streamlined flow of patients into the hospital, but naturally would depend on there being enough flow out as well.

Finally, the problem that should be least prioritised is inpatient transfer prediction. Despite being important, inpatient transfers are generally quite rare due to patients being admitted to appropriate wards from the outset. However, there is value in predicting resource flow and patient movement in order to plan that resource.

### 9.2. Current challenges

#### 9.2.1. Data challenges

Throughout this review, we have emphasised the need for a common dataset that all researchers can use to benchmark their models and experiments on, as well as have agreed definitions of what age ranges ‘elderly’ patient fit amongst other definitions. However, creating a publicly available dataset does not come without its own challenges. The first issue is that of patient privacy. While there are many data anonymisation methods that can be used to remove association of the data with individuals, prior information such as the source hospital can be used to reconstruct the identities of the patients. There then exists a trade-off between how much information is hidden and how useful the data is to machine learning practitioners. A potential solution for this is sourcing data from multiple medical centres and compiling them together in a dataset. This brings us onto the second challenge which is a lack of standardisation in the recording of health data. In order to take advantage of the data from the EHRs from multiple hospitals, we must first stipulate that these hospitals record data in an agreed fashion.

One example of a publicly available healthcare dataset used for benchmarking is MIMIC-III (Johnson *et al* 2016). The success of this dataset can be seen through the volume of works that have used it for model comparison. However, for the purposes of patient flow, this dataset is difficult to use due to its focus on intensive care patients. It therefore does not include the data from the EHRs on the key resource utilisation and patient flows in the hospital (unless they are between intensive care units). A dataset built in a similar fashion to MIMIC-III but with the appropriate patient flow data would benefit the research community greatly.

#### 9.2.2. Technical challenges

Currently the majority of patient flow models use a specific dataset from a hospital that can be derived from a certain subset of patients. The model is then applied to aid that hospital in prediction with very few researchers extending their models beyond their own hospitals. This approach is limited due to the variable and dynamic nature of healthcare datasets. Distributions from the same source hospital are subject to issues such as covariate shift whereby the underlying distributions of the features change with time. Examples are the changes to the distributions that can be found in the EHRs of hospitals during flu season or during the COVID-19 pandemic that has swept the world.

Variability also exists across health care delivery institutions and organisations ranging from small primary care centres to large tertiary hospitals. These organisations are different in their resources, organisational structure, staff training, and culture. These differences create variability in healthcare delivery practices, organisational processes, and patient flow across these different institutions as well as variability in what data is recorded and in what format it is recorded.

Differences also exist in the distributions recorded by healthcare institutions due to the differences in populations across the world. Examples include the prevalence of different diseases across different communities and geographical contexts (e.g. the presence of type II diabetes mellitus can vary from 3.5% to over 20% across different populations) (World Health Organization 2016, James *et al* 2018).

Another issue that is faced is the lack of complete information delivered by the majority of prediction algorithms. While it is useful to know that a patient will be admitted to a certain location in the hospital, having some knowledge of their severity or the likely medications that will be needed for them will further help with the planning of their stay.

These issues faced during deployment create challenges in the applications of machine learning, particularly in the generalisation of models to other hospitals and for their continual use over long periods of time.

In the face of these challenges, we believe that certain research directions will aid future researchers to prepare models that will better serve hospitals to improve patient flow. These research directions should address the issues discussed above, as well as ensure that they integrate seamlessly into the running of the hospital.

### 9.3. Feature engineering

The majority of studies discussed in this review take advantage of the fact that there exist EHR systems in many modern hospitals which allow data extraction and dataset creation. However, there remain challenges in terms of data collection for the different tasks at hand.

The ED admission prediction relies on seasonal information which can be correlated with admissions but is generally a difficult prediction to make. Wearable sensors could benefit this prediction greatly, providing more granular information to the hospital. The sensors could also be provided to patients who need them most (and are most likely to be brought to the ED in an emergency such as elderly patients in care homes).

We believe that further improvements to data collection could be made in the inpatient journey as well as in discharge prediction. Currently, while scans in the hospital are logged on the EHR, the movement of patients to scans are not and nor is the resource associated in moving that patient. These data would be very helpful to provide a more complete picture of what resource each hospitalised patient utilises and thereby helping machine learning scientists create more accurate predictions of the likely resource needed.

In discharge prediction, one of the challenges is that it is generally not recorded when a patient is medically ready to leave the hospital but when they actually do. Augmenting a dataset with this information could help predict when a patient is ready to leave hospital and in doing so, allow the team looking after them to move their resource to more vital care, with a more generalised team looking after the patient thereafter until discharge.

### 9.4. Multitask learning

The first research direction to be considered is multitask learning, a machine learning method that allows multiple tasks to be learned at the same time. One of the aims is to exploit the learning signals generated by training on one task to create an inductive bias in the model that will allow the effective learning of another task by the same model (Caruana 1997).

Multitask learning can be applied to different problems across the four domains of patient flow to both related (e.g. predicting risks of various in-hospital complications) or unrelated tasks (e.g. predicting length of stay in the ED and predicting hospital admission destination). Once again this relates to the usefulness of having more granular information for clinicians to work with. An example may be when predicting the location of admission of a patient to hospital, also having some prediction of whether the patient is likely to deteriorate or not. This gives better indications of the likely resource requirement of the patient as well as their likely trajectory within the hospital. While this could be done using separate models for each prediction, a single model that can embed an accurate representation of the patient will be more informative and useful to clinical staff. As a result, a key component of this work will be in the development of representation learning algorithms (Bengio *et al* 2013, Van Den Oord *et al* 2017) that are capable of representing patient conditions upon presentation to the ED or admission to hospital.

Multitask learning has been applied in many healthcare applications to leverage the shared information across different tasks. Huang and Dong (2018) have used multitask learning to predict major adverse cardiac events, identifying each type of adverse event as a single task as opposed to having a multiclass classification. Xia *et al* (2019) have also used this approach to predict prescription patterns for various drugs that are given to similar patients. Multi-task learning has also been used in medical imaging. Khosravan *et al* (2019) have used multitask learning in the detection of abnormal nodules on chest CT scans for lung cancer screening. They jointly train their model to segmenting potential abnormalities and identify the presence of a nodule in the region of interest. This is further evidence of how more granular information from the model can provide clinicians with better insights into the condition of the patient.

### 9.5. Transfer learning

Transfer learning is based on the principle of knowledge transfer across different machine learning tasks and models. It is based on the notion that knowledge gained by the algorithm when trained to solve a particular problem can be stored and applied to solve another related problem, which means it is closely related to multitask learning. This approach includes transferring knowledge from the source domain, *D*_*S*_, to the target domain, *D*_*T*_, to help improve the learning of the target-domain task, *T*_*T*_.

Transfer learning can provide significant advantages in the applications of machine learning in patient flow. It can enable (a) the transfer of knowledge across different tasks and (b) the transfer of knowledge across different populations. The former can help overcome the lack of clinical data for certain problems. For example, one of the barriers to developing effective machine learning tools for COVID-19 patients is the lack of data on COVID-19 patients. A transfer learning approach can provide a solution by using a model that is pretrained on a large non-COVID-19 dataset and adapting it to perform the task of interest in COVID-19 patients. Transfer learning has been used to overcome the lack of COVID-19 imaging data by Mahmud *et al* (2020). They trained a convolutional neural network by using a pretrained a neural network (pretrained on a dataset of bacterial and viral pneumonia chest x-ray scans) and fine-tuned this using scans from COVID-19 patients. This was done due to the scarce availability of chest x-rays from these patients.

Transfer learning can also help us transfer knowledge across different populations. This is valuable clinically given the diversity and differences in the genetic predispositions, prevalence of diseases, lifestyles, and risk factors across different populations. Mao *et al* (2018) used transfer learning to generalise their sepsis prediction algorithm to a new healthcare setting. They trained their prediction model using data from the MIMIC-III dataset (data from ICU patients) and transferred the model to a dataset from the University of California, San Francisco (UCSF) Medical Centre (a dataset of in-hospital patients from a variety of specialty wards). Their transfer learning approach was based on adding incremental amounts of data from the UCSF dataset to the MIMIC training dataset, resulting in better generalisation of the model to the dataset being introduced.

Transfer learning represents an interesting target for future research in patient flow machine learning applications. Transfer learning can be used to generalise models across different healthcare contexts and to overcome a lack of recorded data.

### 9.6. Continual learning

Continual or lifelong learning refers to the ability to continually learn over time by accommodating new knowledge while retaining previously learned experiences. This approach has the potential to enable machine learning models in the healthcare space to adapt and adjust automatically to new context and settings like a new healthcare context, new patient population, or a new and emerging disease. This has the potential to enable the creation of dynamic clinical AI models that optimise clinical management decision in real time and learn from the continuous influx of information in real world healthcare context. A continually learning algorithm should be an adaptive algorithm capable of learning from a continuous stream of information, with such information becoming progressively available over time. The accommodation of new information should occur without catastrophic forgetting or interference (Parisi *et al* 2019).

However, continual learning represents a long-standing challenge due to the susceptibility of machine learning models to catastrophic forgetting. This phenomenon refers to the decrease in model performance or the complete overwriting of the previously learned information when new knowledge is introduced.

A paper published in the Lancet in 2020 (Lee and Lee 2020b) highlights the promise of continual learning in revolutionising the applications of clinical AI and leveraging the continuous influx of clinical information to improve patient care. Shah *et al* (2019) highlight that machine learning algorithms that are capable of continuous learning are a critical future research and translational direction in healthcare AI. They also report that the FDA is considering widening its regulatory framework to include AI-based Software as Medical Device (SaMD) systems that are capable of continuously learning and optimising performance in real-time to improve patient care.

Continual learning promises considerable value in patient flow as it would enable machine learning models to adjust to different healthcare settings continuously and automatically. Therefore machine learning algorithms would be able to absorb the variation across different healthcare institutions and patient populations. Moreover, continual learning may enable machine learning algorithms to continuously learn after deployment to clinical settings gradually improving their performance through use.

## 10. Conclusion

We have seen in this review that machine learning in patient flow is a vast if disjoint field. There are many works published with the majority focused on the hospital associated with the authors and little by way of comparison to other hospitals or works. We therefore propose the introduction of a publicly available dataset based on the electronic health records of a given hospital. This should include enough information on all four subcategories of the patient flow process (as highlighted previously) and crucially, must have strict definitions for patient types. The dataset should include:

• Seasonal information such as the weather, national holidays and ideally EHR data from multiple hospitals.
• Strict definitions of what age ranges ‘elderly’ or ‘young’ patients fall into for reproducibility and model validation.
• Pre-defined tasks such as ‘prediction of patient transfer in 3 h from time of measurement’. By creating these pre-defined tasks we improve the ability of researchers to benchmark against each others work and develop upon each others models.
• A standardised definition of co-morbidities in patients.

We believe that in creating this dataset, a culture of benchmarking on the dataset can be created thereby encouraging researchers to compare their models, build more sophisticated models based on previously published work and crucially provide some external validation to the trained models.

## Figures and Tables

**Figure A1.**
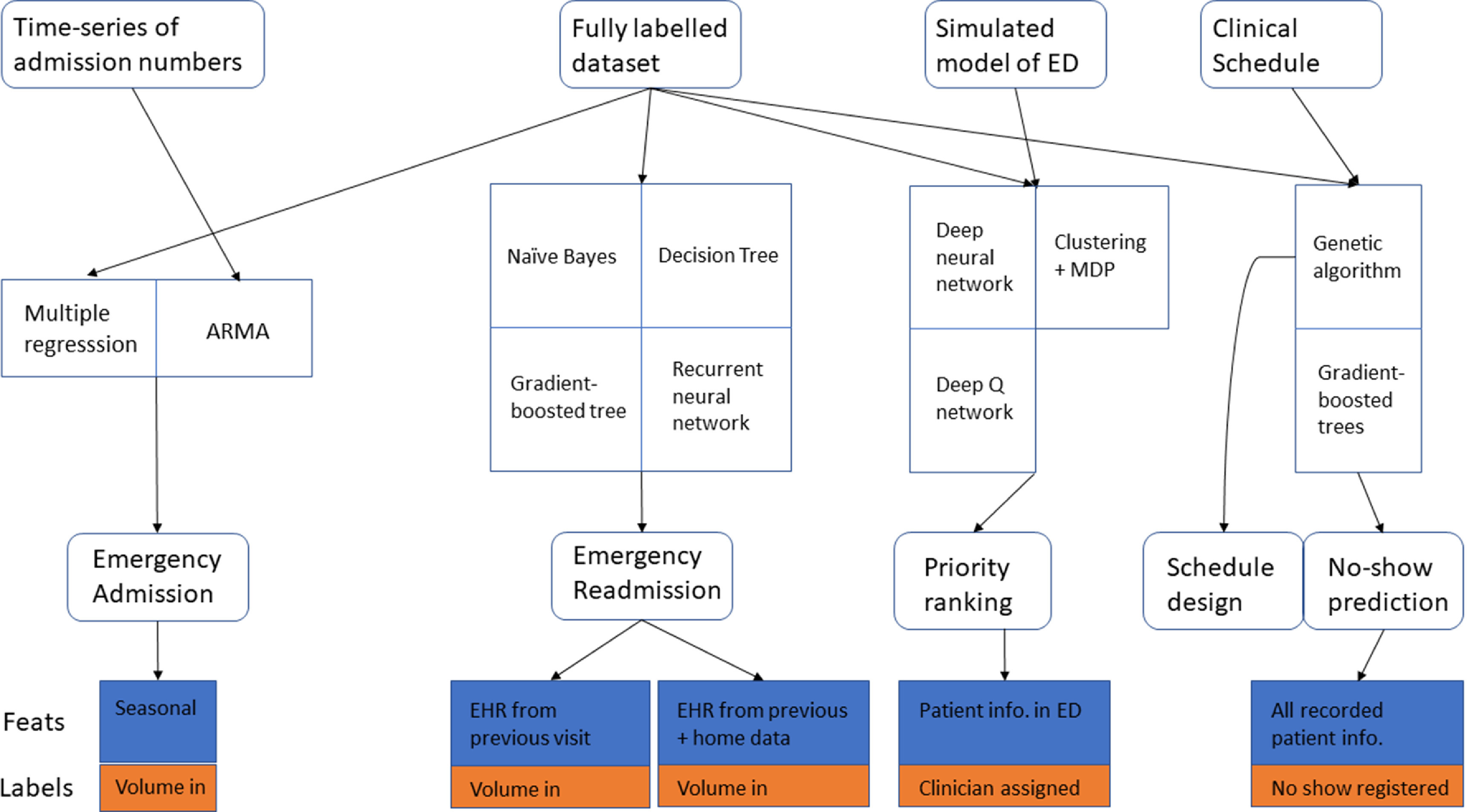
A flowchart showing the models that have been used for the separate prediction problems for predicting ED admissions. The top row shows the sources of data used, the row below shows the models that have been used in various different works for the prediction problem, below this is the problem being tackled and below each of these problems is one of the datasets used in a study to train the model.

**Figure A2.**
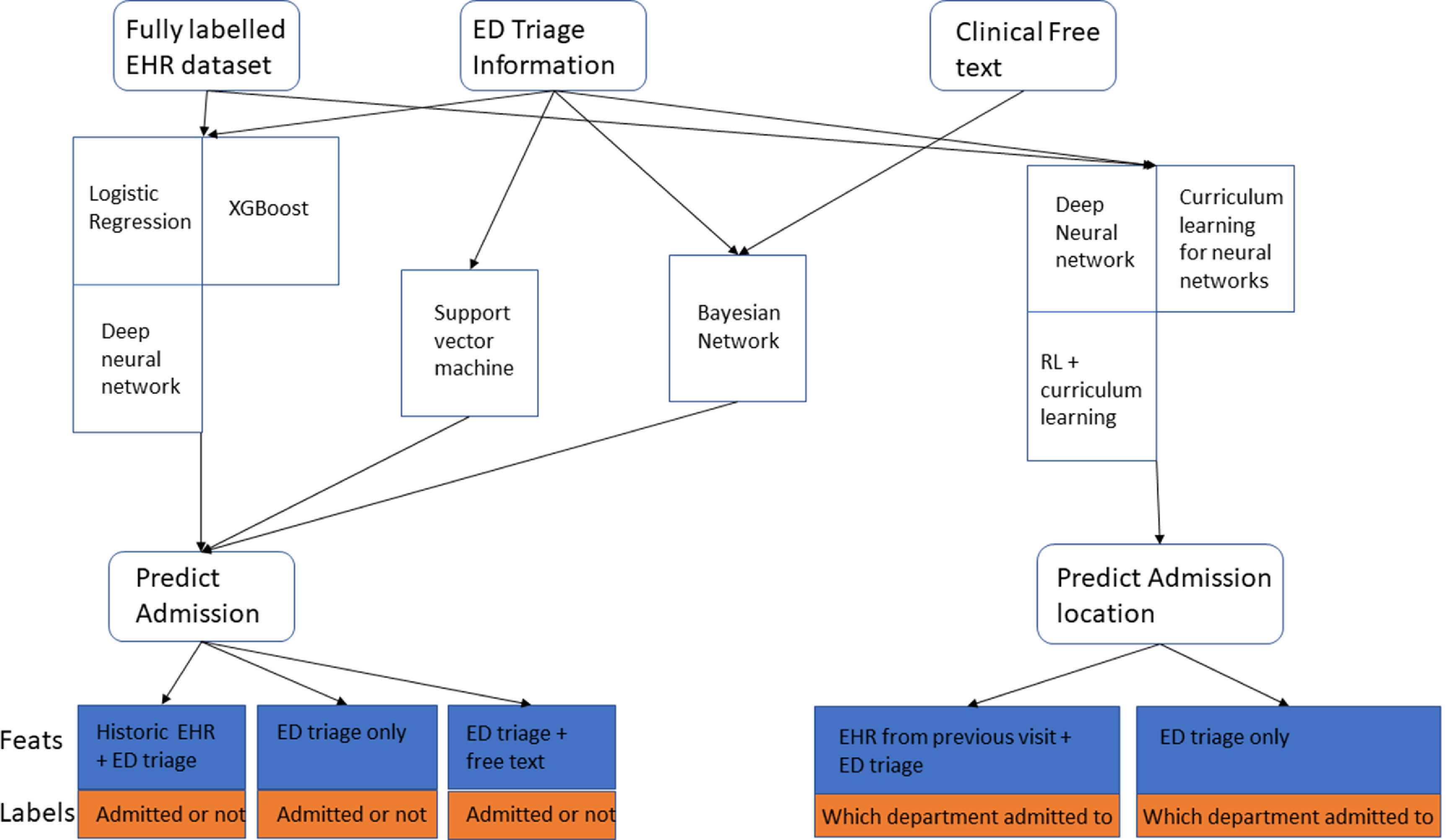
A flowchart showing the models that have been used for the separate prediction problems for predicting ED to inpatient admissions. The top row shows the sources of data used, the row below shows the models that have been used in various different works for the prediction problem, below this is the problem being tackled and below each of these problems is one of the datasets used in a study to train the model.

**Figure A3.**
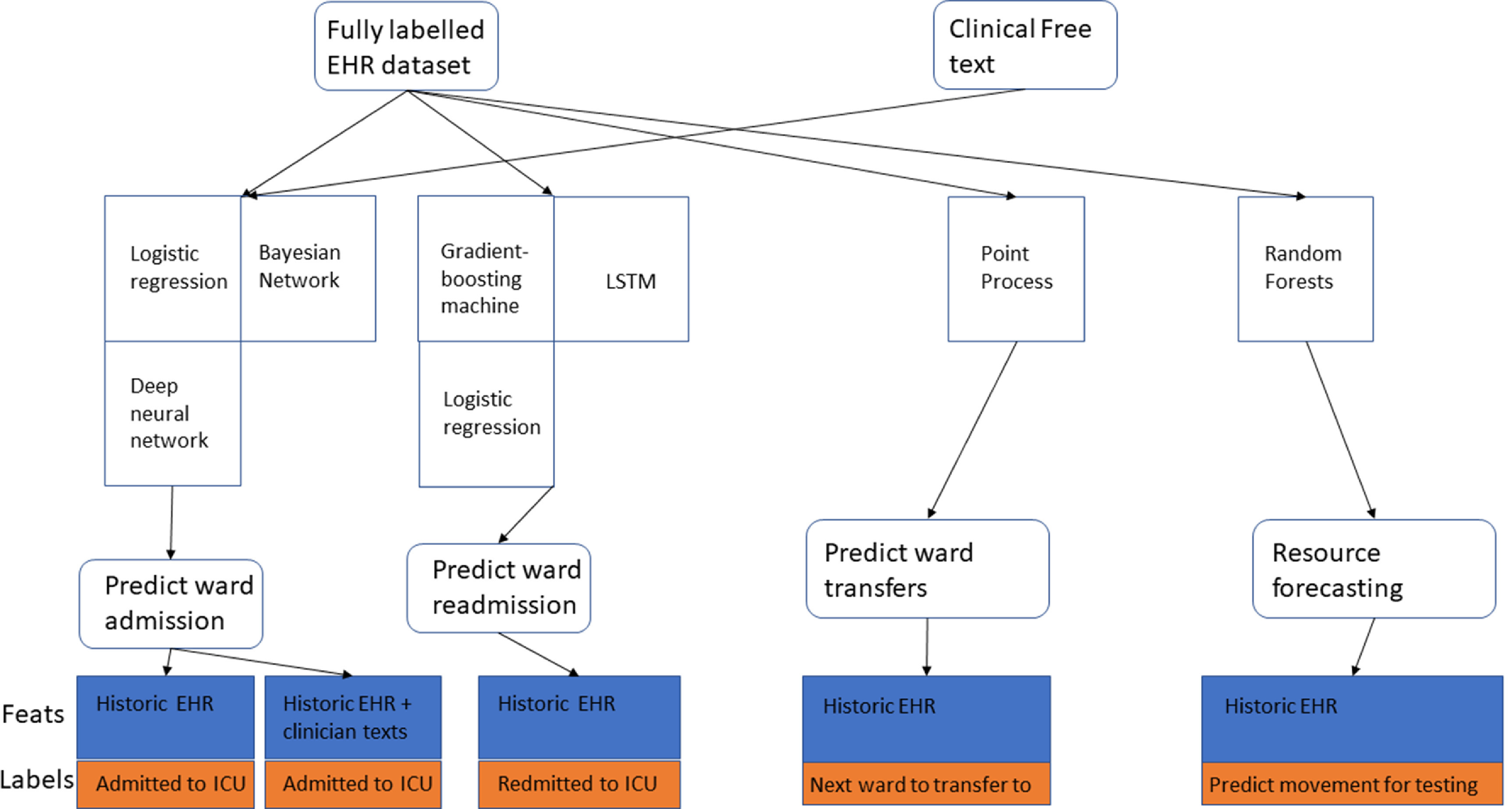
A flowchart showing the models that have been used for the separate prediction problems for predicting inpatient transfers. The top row shows the sources of data used, the row below shows the models that have been used in various different works for the prediction problem, below this is the problem being tackled and below each of these problems is one of the datasets used in a study to train the model.

**Figure A4.**
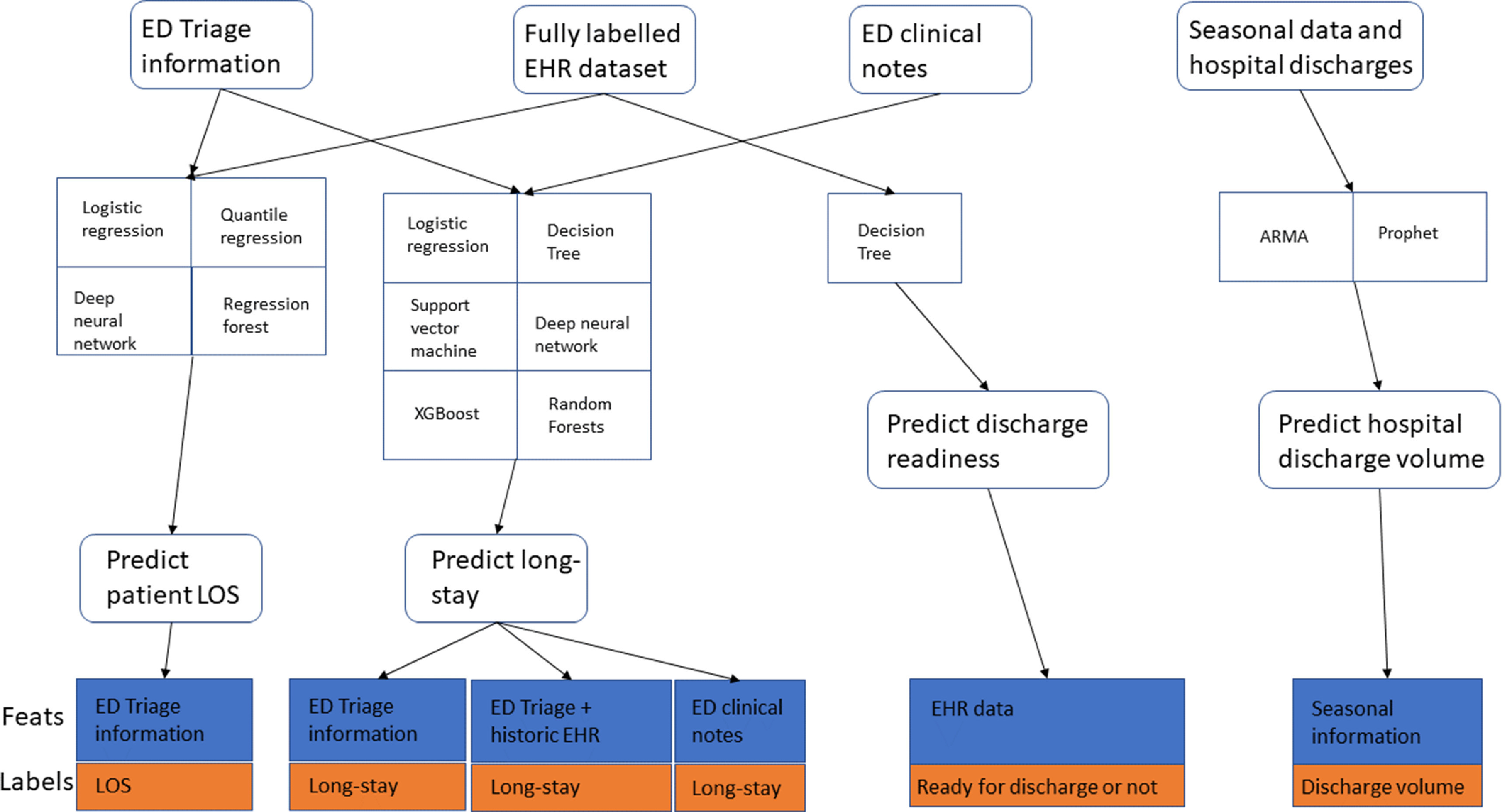
A flowchart showing the models that have been used for the separate prediction problems for predicting discharges. The top row shows the sources of data used, the row below shows the models that have been used in various different works for the prediction problem, below this is the problem being tackled and below each of these problems is one of the datasets used in a study to train the model.

## Appendix Models used for the prediction problems

In order to provide a more complete picture of the works that have been conducted in the space of machine learning in patient flow we here provide flow charts including the models and datasets that have been used to make the predictions. Figure A1 corresponds to ED admissions, figure A2 corresponds to the ED-inpatient interface, figure A3 corresponds to inpatient transfers and figure A4 corresponds to discharge.
